# Beyond traditional biopsies: the emerging role of ctDNA and MRD on breast cancer diagnosis and treatment

**DOI:** 10.1007/s12672-025-01940-6

**Published:** 2025-03-06

**Authors:** Hussein Sabit, Manar G. Attia, Nouran Mohamed, Pancé S. Taha, Nehal Ahmed, Salma Osama, Shaimaa Abdel-Ghany

**Affiliations:** 1 Department of Medical Biotechnology, College of Biotechnology, Misr University for Science and Technology, P. O. Box 77, Giza, Egypt; 2 Department of Pharmaceutical Biotechnology, College of Biotechnology, Misr University for Science and Technology, P. O. Box 77, Giza, Egypt; 3 Department of Environmental Biotechnology, College of Biotechnology, Misr University for Science and Technology, P. O. Box 77, Giza, Egypt; 4 Department of Agriculture Biotechnology, College of Biotechnology, Misr University for Science and Technology, P. O. Box 77, Giza, Egypt

**Keywords:** Breast cancer, BC, ctDNA, MRD, Personalized medicine, Early detection

## Abstract

Breast cancer management has traditionally relied on tissue biopsies and imaging, which offer limited insights into the disease. However, the discovery of circulating tumor DNA (ctDNA) and minimal residual disease (MRD) detection has revolutionized our approach to breast cancer. ctDNA, which is fragmented tumor DNA found in the bloodstream, provides a minimally invasive way to understand the tumor's genomic landscape, revealing heterogeneity and critical mutations that biopsies may miss. MRD, which indicates cancer cells that remain after treatment, can now be detected using ctDNA and other advanced methods, improving our ability to predict disease recurrence. This allows for personalized adjuvant therapies based on individual MRD levels, avoiding unnecessary treatments for patients with low MRD. This review discusses how ctDNA and MRD represent a paradigm shift towards personalized, genomically guided cancer care, which has the potential to significantly improve patient outcomes in breast cancer.

## Introduction

Despite advances in breast cancer (BC) detection, prevention, and treatment, over 2.3 million new cases occur annually, with over 650,000 deaths [1]. Tissue biopsies, while crucial, are invasive and limited in scope, capturing only a snapshot of a potentially dynamic tumor [2]. Liquid biopsy, which involves analyzing circulating tumor DNA (ctDNA), is a powerful tool that complements tissue biopsies and provides a dynamic view of BC [3]. Driven by breakthroughs in ctDNA detection methods, such as NGS, research on this topic has surged in the past decade. ctDNA levels are strongly correlated with tumor burden, prognosis, stage, and treatment response [4, 5]. Landmark studies across various cancers highlight the pivotal role of ctDNA in identifying minimal residual disease (MRD) and diagnosing molecular relapse, even after seemingly successful treatment [6]. MRD refers to any lingering molecular trace of cancer that is often detectable after surgery or definitive therapy.

This review delves into the immense potential of ctDNA-based MRD detection to guide treatment decisions in both adjuvant and neoadjuvant settings, thereby optimizing treatment efficacy. It also explores the evidence supporting various strategies for integrating ctDNA into clinical trial designs.

## BC molecular subtypes

**Fig. 1:**
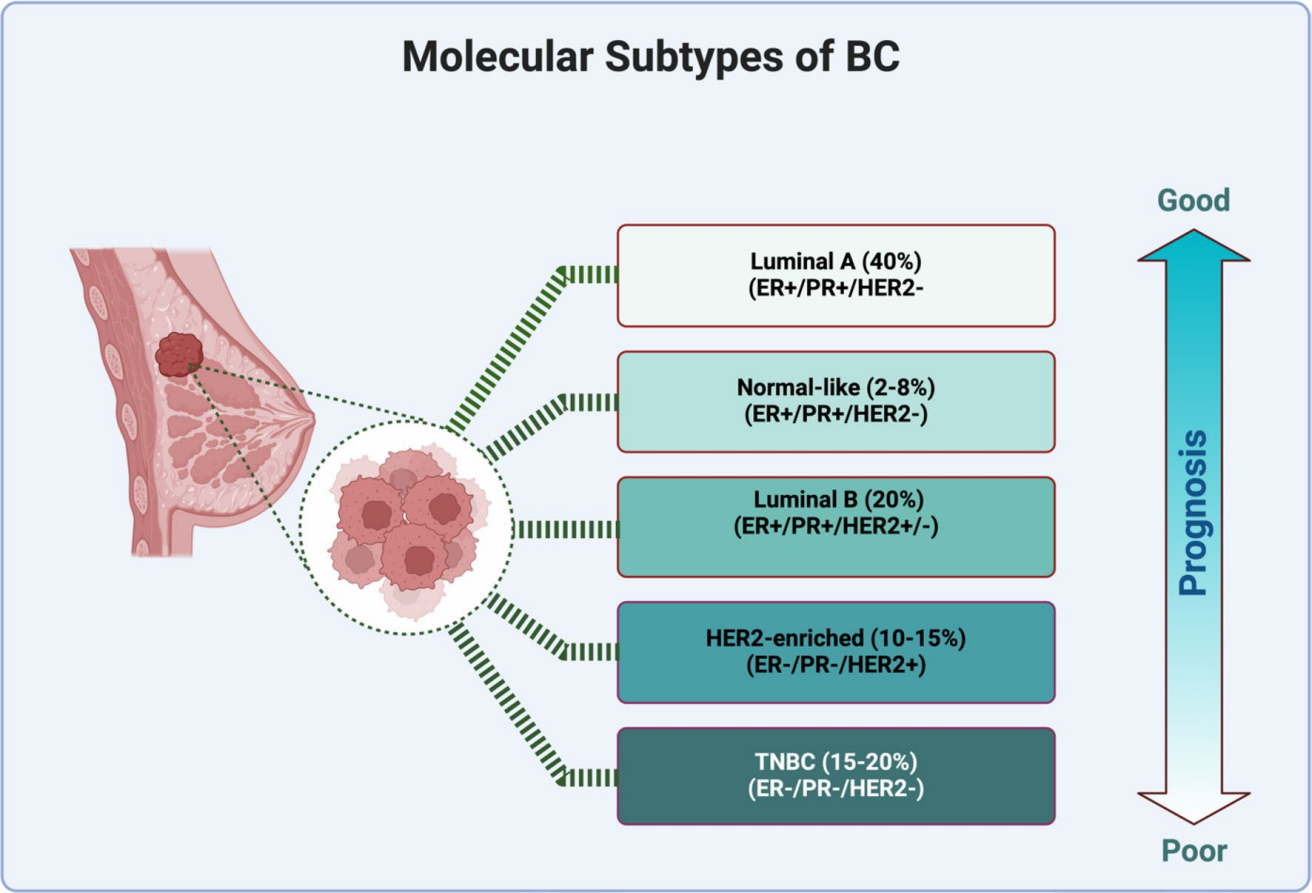
The molecular subtypes of BC. These subtypes are categorized according to the expression of ER, PR, and HER2, along with other markers such as cytokeratin and Ki67. Created with BioRender

At the molecular level, several subtypes of BC exist, making it a heterogeneous disease. It is characterized by high genomic instability. Defects in telomere maintenance, DNA replication, transcription, damage repair, and mitotic chromosome segregation cause genomic instability [7]. This heterogeneous disease can be classified into five molecular subtypes, luminal A, luminal B, human epidermal growth factor receptor 2 (HER2)-positive, basal-like, and normal-like BC, according to the expression of the estrogen receptor (ER) progesterone receptor (PR), the overexpression of HER2 cytokeratin CK, and the proliferation index Ki67 (Fig. 1). The classification of BC may be improved by including a variety of molecular markers, such as miRNAs, and mutations in genes such as *P53*, *BRCA1*, and *BRCA2* [8]

### Luminal subtypes

Approximately 60% of BC cases are classified as luminal type, which is distinguished by ER expression [9]. Most luminal tumors express PR, ER, and other genes associated with ER activation. The luminal subtype is classified into two subgroups: A and B [10].

#### Luminal A

Tumors are defined by the presence of ER + , high expression of PR and HER2-, low expression of Ki-67, and high expression of CK. Clinically, they include a wide range of low histological grades and slow growth. They have been associated with a good prognosis, a low incidence rate and a high survival rate and generally show less lymph node involvement. These carcinomas exhibit limited benefit from chemotherapy and a high rate of hormone therapy response to tamoxifen or aromatase inhibitors [11, 12].

#### Luminal B

Luminal B tumors are characterized by increased expression of proliferative and cell cycle genes, decreased expression of PR, decreased expression of CK, and increased expression of genes that encode two immunohistochemical (IHC) markers of cell proliferation (Ki-67) and proliferating cell nuclear antigen. It represents 10–20% of luminal tumors. This subtype can be classified as Luminal B (HER−), defined by the presence of ER + , PR−, HER2−, and high levels of Ki-67, or Luminal B (HER2 +), defined by the presence of ER + , HER2 + , and any level of PR and Ki-67 [13]. These tumors have a worse prognosis, grow faster and low survival rate than luminal A tumors because of their elevated Ki-67. Unlike luminal A tumors, luminal B tumors are associated with a high frequency of *p53* mutations [14]. These tumors are the most aggressive form of hormone-dependent BC and require additional therapy, such as chemotherapy [15].

#### Triple-negative BCs (TNBC)

TNBC (*aka* basal-like BC (BLBC)) is a type of tumor characterized by the absence of ER, PR, and HER2. TNBC accounts for approximately 20% of all BC cases. The remaining TNBCs can be divided into six subgroups: basal-like 1 (BL1), basal-like 2 (BL2), mesenchymal, mesenchymal stem-like (MSL), luminal androgen receptor (LAR), and immunomodulatory (IM) [16]. Approximately 70–80% of all TNBCs are of the basal-like subtype. Basal-like BC is characterized by high expression of proliferation-related genes, integrin B4, laminin, and keratin 17 (a type I cytokeratin).

Chemotherapy remains the mainstay of treatment for both adjuvant and neoadjuvant therapy in basal-like TNBC patients due to the lack of targeted therapies based on ER, PR, and HER2 expression; however, other options are available. These include PARP inhibitors (for tumors with BRCA mutations) and immunotherapy (for PD-L1-positive tumors) [17].

#### HER2 subtype

Human epidermal growth factor receptor 2 (HER2) encodes the transmembrane tyrosine kinase receptor. The HER2 proto-oncogene encodes the HER2 receptor located on chromosome 17q21 in the absence of ER and PR [18]. Approximately 12–20% of all BCs are classified as HER2-positive, meaning that they either overexpress the HER2 protein or have an amplified HER2 gene. This leads to aggressive tumor growth and poor prognosis compared to luminal tumors [19]. This subtype can be classified into two subgroups: HER2−enriched, characterized by the expression of HER2 + , E−, PR−, and Ki67, and luminal HER2, characterized by the expression of E + , PR + , HER2 + , and Ki-67 [9]. HER2-overexpressing tumors were diagnosed by fluorescence in situ hybridization (FISH), which shows HER2 gene amplification, and immunohistochemistry (IHC), which reveals HER2 overexpression [20]. HER2 tumors react favorably to trastuzumab, a humanized monoclonal antagonist, and are sensitive to traditional chemotherapy [21].

#### Normal-like breast cancer

Normal-like BCs typically exhibit characteristics similar to those of luminal A BCs, although there are minor distinctions in their overall genetic makeup. Both normal-like and luminal A tumors commonly display classical immunohistochemistry markers such as ER + , PR + , HER2 − , and low KI-67 expression [22]. The normal-like breast subtype presents as a triple-negative tumor with a molecular profile similar to that of basal-like carcinoma. However, compared with basal-like carcinoma, normal-like breast carcinoma has a more favorable prognosis [23].

#### Claudin-low breast cancer subtypes

In addition to the five major breast cancer molecular subtypes, another subtype displaying a different profile was identified, namely, the claudin-low breast cancer subtype. It is the least characterized subtype in the literature compared to other molecular subtypes, though it accounts for 7–14% of all invasive breast cancers [24]. This subtype is characterized by low expression of genes associated with tight junction proteins, such as claudin-3, claudin 4, claudin 7, and E-cadherin, as well as poor survival and prognosis [24, 25]. With respect to hormone receptor status, these tumors preferentially display a triple-negative (TN) phenotype. However, the proportion of patients with a TN phenotype in these tumors is smaller than that in basal tumors [24]. These tumors exhibit high genomic instability, with numerous gains and losses, similar to those of basal tumors. Additionally, claudin-low tumors exhibit lower expression of luminal epithelial and cell–cell adhesion markers and a greater prevalence of markers for epithelial-to-mesenchymal transition and immune response genes (lymphocyte markers). The tumors also display cancer stem cell-like traits and are less differentiated [26]. These cells also exhibit increased immune and stromal cell infiltration [27]. There is increased activity in the estrogen receptor (ER), progesterone receptor (PR), EGFR, SRC, and TGFβ pathways but decreased activity in the MYC and PI3K pathways [24]. The prognostic features of claudin-low tumors are more similar to those of luminal A tumors—the response rate to standard preoperative chemotherapy falls between that observed for basal-like and luminal tumors [26].

## Molecular pathways

### PI3K/AKT/mTOR signaling pathway

**Fig. 2:**
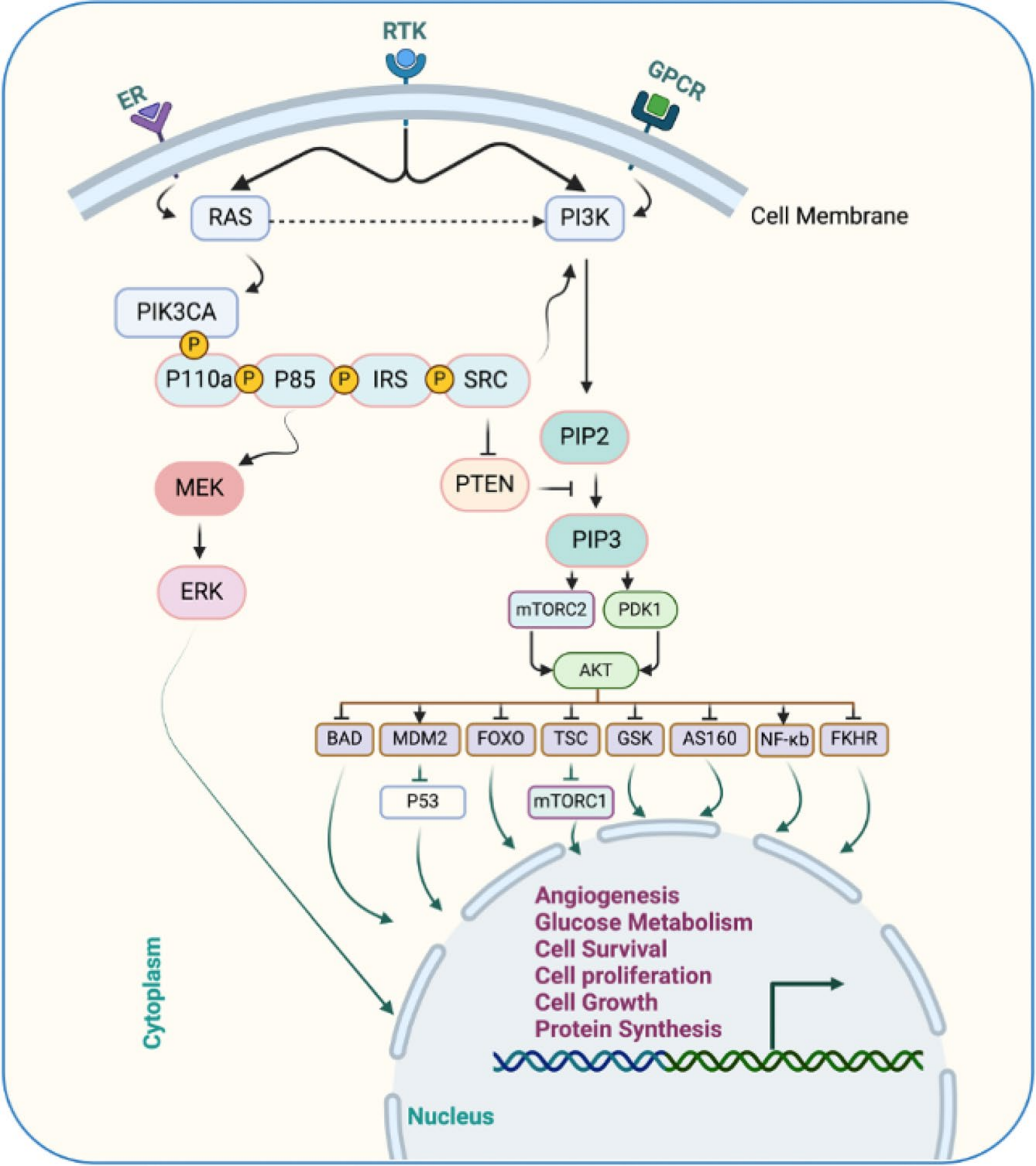
The molecular pathway of PI3K/AKT/mTOR. The ER and RTK play a role in initiating the cascade of activation of several proteins, promoting cell proliferation, growth, and survival. AKT: Protein kinase B, AS: Akt substrate, BAD: Bcl2 antagonist of cell death, ER: Estrogen receptor, ERK: Extracellular-signal regulated kinase, FKHR: Forkhead transcription factor, FOXO: Forkhead Box O, GPCR: G protein-coupled receptor, GSK: Glycogen synthase kinase, IRS: Insulin receptor substrate, MDM: Mouse double minute, MEK: Mitogen-activated protein kinase, mTOR: Mammalian target of rapamycin, NFκB: Nuclear factor-κb, PDK: Pyruvate dehydrogenase lipoamide kinase isozyme, PI3K: Phosphatidylinositol 3-kinase, PIK3CA: Phosphatidylinositol-4,5-Bisphosphate 3-Kinase Catalytic Subunit Alpha, PIP: Phosphatidylinositol phosphate, PTEN: Phosphatase and tensin homolog, RTKs: Receptor tyrosine kinases, SRC: Rous sarcoma, TSC: Tuberous sclerosis complex. Created with BioRender

Extracellular signaling pathways are crucial for many vital cellular processes, such as angiogenesis, cell growth, cell metabolism, apoptosis, and cell proliferation. They produce molecules that attach to other cell receptors and initiate intracellular signaling pathways (Fig. 2). In this way, cells respond and adapt to changes [28]. Within the intricate network of cellular signaling pathways, ligand binding to specific receptors initiates a cascade of events akin to coordinated sympathy.

Phosphatidylinositol 3-kinase (PI3K), a key enzyme, catalyzes the phosphorylation of phosphatidylinositol (4,5)-bisphosphate (PIP2) to phosphatidylinositol (3,4,5)-trisphosphate (PIP3). This lipid molecule acts as a signaling cue, recruiting the protein kinases Akt and phosphoinositide-dependent protein kinase 1 (PDK1) to the plasma membrane via their pleckstrin homology (PH) domains. Subsequently, mTOR complex 2 (mTORC2) phosphorylates Akt at Ser473, which is primed for further activation by PDK1 at Thr308. Fully activated Akt phosphorylates diverse target proteins both on the cell membrane and within the cytoplasm and nucleus, regulating critical cellular processes such as survival, growth, and proliferation [29].

### PI3K/AKT/mTOR mutations

BC thrives through dysregulation of the PI3K pathway, often fuelled by mutations or amplifications in key genes. Among these genes, PIK3CA, which encodes the p110α catalytic subunit, is the most frequently mutated gene in human cancers. Notably, amplification of PIK3CA has also been observed in BC, further confirming its influence. Studies have reported the highest prevalence of PIK3CA mutations in basal-like and HER2-positive BC subtypes, highlighting their potential role in aggressive forms of the disease. By understanding these genetic alterations and their impact on the PI3K pathway, researchers can pave the way for developing targeted therapies specifically designed to combat these mutations and improve patient outcomes [30]. As a significant boost for personalized BC care, the FDA now allows testing for PIK3CA mutations, which are found in 30–40% of patients, using both tumor tissue and blood-based circulating tumor DNA (ctDNA). These mutations hyperactivate the PI3KA pathway, thus fuelling tumor growth. Notably, 11 mutations in exons 9 and 20 showed strong activation, highlighting their potential importance in guiding targeted therapies and treatment decisions for patients harboring these specific alterations.

This advancement opens doors for earlier detection, more precise diagnoses, and potentially the development of drugs specifically targeting the hyperactive PI3K pathway in patients with these mutations [31]. Oncogenic mutations and the binding of growth factors or insulin to RTKs activate PI3Kα [32]. The PI3K/AKT pathway in BC is triggered by either PIK3CA or AKT1 mutations and PTEN loss [33, 34].

In a sample of 6338 patients, nearly 36% of BC patients harbored mutations in the *PIK3CA* gene, which encodes the p110α subunit of the PI3K pathway. Interestingly, these mutations were not scattered randomly but clustered in three specific "hotspots" within the coding sequence of the gene. Notably, two of these hotspots reside within the helical domain of the p110α protein, while the third resides in its catalytic domain.

These mutations are not mere typos; rather, they are single nucleotide substitutions that lead to significant changes in the encoded amino acids. These altered amino acids trigger a detrimental consequence: a gain of function. This means that the mutated p110α becomes hyperactive, constantly pumping out signals that promote uncontrolled cell growth and, ultimately, tumor formation. Understanding the precise location and functional impact of these hotspot mutations is crucial for developing effective therapies. By targeting the overactive p110α protein or downstream signaling molecules, researchers hope to offer more precise treatment options for patients with *PIK3CA*-driven BC [35]. Compared with those in HER2 patients (42%) and HER2 + patients (31%), PIK3CA mutation rates in TNBC patients were reduced to 16%. Moreover, 28% of *PIK3CA* mutations in circulating tumor DNA have been found in patients with advanced HER2 BC [31]. According to tumor sequencing studies, somatic mutations in *PIK3CA* lead to tumor progression by enhancing *PIK3CA* function [36]. In addition, *PIK3CA* mutations in exons 9 and 20 of human BC have been reported in studies using immortalized breast epithelial cells. *PIK3CA* is the most identified mutation and is related to increased kinase activity in the *PI3K* pathway. *PIK3CA* mutations promote the proliferation and invasion of human cancer cells [37].

p110α, the catalytic subunit of the PI3Kα complex required for normal growth and proliferation, is required for tumor signaling and growth caused by *PIK3CA* mutations or RTKs [32]. p110β promotes tumorigenesis in PTEN-deficient cells. BCs with increased AKT phosphorylation/activation and PTEN loss have poor disease outcomes. Furthermore, the loss of PTEN activity and activation of the PI3K signaling pathway are linked to endocrine therapy resistance [34].

Somatic mutations or deletions in PTEN, a critical tumor suppressor, are frequently observed in breast tumors and disrupt the phosphatase activity of PTEN, leading to unchecked activation of the PI3K/AKT/mTOR pathway. As mentioned above, this pathway regulates cell growth, survival, migration, and genomic stability through a cascade of phosphorylation events. By dampening these signals, PTEN ensures orderly cell cycle progression, promotes apoptosis in damaged cells, and safeguards DNA integrity via repair mechanisms. However, the inactivation of PTEN mutations unleashes the PI3K pathway, driving uncontrolled cell proliferation, migration, and ultimately tumorigenesis. Understanding these molecular underpinnings provides valuable insights for developing targeted therapies aimed at restoring PTEN function or inhibiting the hyperactive PI3K pathway in patients [38]. Luminal BC frequently harbors alterations in the PI3K pathway driven by PIK3CA mutations, PTEN loss, or aberrant downstream protein phosphorylation. Zardavas et al. (2018) analyzed data from over 10,000 patients and revealed that PIK3CA mutations were primarily associated with ER-positive tumors in 32% of the patients. Interestingly, the frequency of these mutations increased with age, tumor grade, and tumor size. Moreover, a distinct distribution across BC subtypes was observed: 18% were ER-negative/HER2-negative, 22% were HER2-positive, and 37% were ER-positive/HER2-negative, highlighting their potential role in this aggressive subtype [39].

Deng et al. investigated the prevalence of PI3K pathway alterations in BC by analyzing tumors from 507 patients at the West China Hospital (2008–2013). They identified 3% of patients with ER + /PR + /HER2-positive tumors harboring AKT1 mutations, suggesting a potential role for this specific subtype. Notably, the present study revealed a much greater prevalence of PIK3CA mutations (46.5%), particularly within the same ER + /PR + /HER2-positive group. Interestingly, 35 patients harbored multiple PIK3CA gene variants, raising questions regarding the potential synergistic effects of cooccurring mutations in this pathway [40].

PIK3CA mutations, frequently involving hotspot single amino acid substitutions within the kinase domain, are recognized as oncogenic drivers in various cancers, including BC. Notably, in primary and metastatic ER + /HER2-positive breast tumors, the incidence of PIK3CA mutations is 40%, highlighting its crucial role in this subtype. This widespread presence makes PIK3CA an attractive therapeutic target. Anderson et al. reported a 36% prevalence of PIK3CA mutations in HR + /HER2-negative metastatic BC, suggesting potential broader therapeutic applicability [31, 41, 42].

In addition to PIK3CA, the PI3K pathway harbors diverse alterations in BC, including mutations in receptor tyrosine kinases (RTKs), such as HER2 (ERBB2) and fibroblast growth factor receptor (FGFR)1, as well as in the AKT1 and PTEN genes. These genetic changes often occur in concert; ligand binding to RTKs or G protein-coupled receptors (GPCRs) triggers PI3K activation, while PIK3CA mutations can cooccur with PTEN loss or HER2 overexpression, further amplifying pathway signaling [28].

Highly clonal PIK3CA mutations were predominantly found in TNBC subtypes exhibiting luminal phenotypes and androgen receptor expression (40%), whereas a significantly lower percentage (4%) of TNBC patients lacking androgen receptors exhibited these mutations. This finding suggests a potential role for PIK3CA mutations in shaping luminal-like features in TNBC.

In the BC cancer landscape, HER2 overexpression, which is found in 15–20% of cases, is associated with aggressive behavior. Interestingly, PIK3CA mutations were prevalent in luminal A tumors (45%), highlighting their relevance in this subtype. However, their prevalence declines in aggressive subtypes: 4% in tumors with AKT1/PTEN mutations and only 7% in basal-like tumors [43–45].

## Liquid biopsy

Liquid biopsy by circulating tumor DNA (ctDNA) analysis offers a promising alternative to traditional tissue biopsy methods for detecting PIK3CA mutations, thereby enhancing minimally invasive patient monitoring and treatment selection. However, it is crucial to acknowledge the potential limitations of ctDNA testing, including varying sensitivity across technologies and tumor types. Additionally, the quantity and dynamic nature of ctDNA can influence its correlation with patient outcomes. Therefore, a comprehensive understanding of these limitations and continual validation of ctDNA testing methods are crucial for ensuring their optimal use in guiding personalized cancer treatment strategies [46–48].

A study conducted by Dumbrava et al. aimed to determine whether PIK3CA mutations could be detected in low-volume plasma ctDNA from patients with advanced cancers using droplet digital PCR (ddPCR). Furthermore, we investigated the association between the quantity and dynamic changes in ctDNA, as measured by the variant allele frequency (VAF) of PIK3CA mutations, and patient outcomes. In their study of 68 patients with advanced cancers, the authors reported an 85% detection rate of PIK3CA mutations in tumor tissue and 74% in circulating tumor DNA (ctDNA). Overall, there was 72% concordance between these two detection methods. Further analysis excluding samples from 26 patients without evidence of disease progression revealed a significantly higher concordance rate of 91%. This suggests that ctDNA detection might be more reliable in the context of active disease. Interestingly, patients with a ctDNA variant allele frequency (VAF) of PIK3CA mutations of approximately 8.5% exhibited longer median survival than those with higher VAFs. Additionally, longitudinal ctDNA analysis of 18 patients revealed that patients who experienced a decrease in the PIK3CA VAF over time had a longer time to treatment failure (TTF) than patients with stable or increasing VAFs [49].

**Fig. 3:**
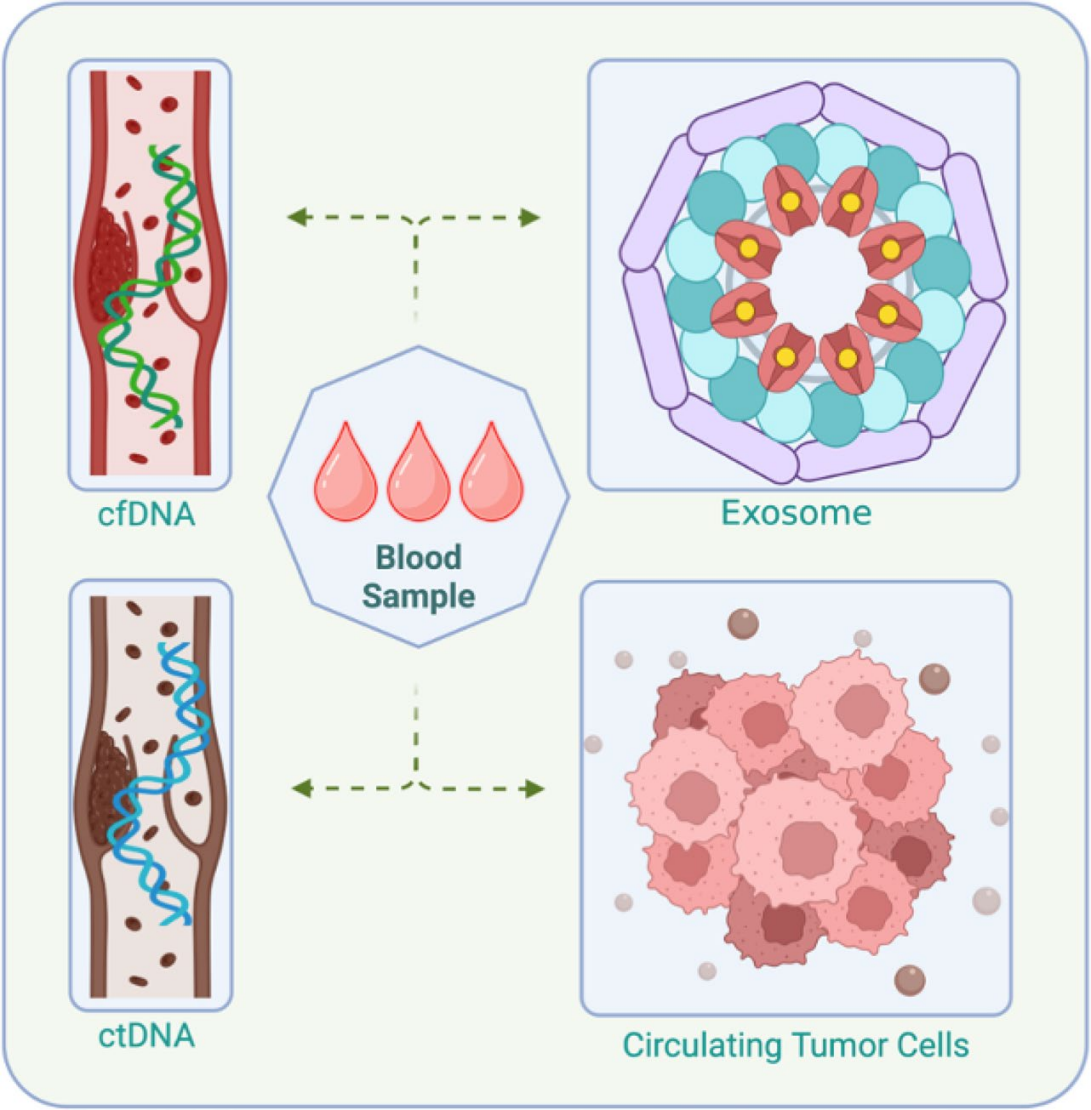
Circulating molecules that could be used in diagnosing cancer, including ctDNA, cfDNA, circulating tumor cells, and exosomes. cfDNA: cell-free DNA, ctDNA: circulating tumor DNA. Created with BioRender

Liquid biopsy offers a revolutionary approach to cancer analysis, replacing invasive tissue biopsies with a simple blood draw or other minimally invasive sample collection methods. This technique harnesses the presence of circulating tumor cells (CTCs), cell-free tumor DNA (ctDNA), and other tumor-derived molecules in bodily fluids such as blood, urine, or cerebrospinal fluid tumors [50]. (Fig. 3). By analyzing these liquid tumor biopsies, researchers have gained valuable insights into tumor biology and heterogeneity and the presence of diverse populations within a single tumor [51]. This heterogeneity arises from a complex interplay of genetic and epigenetic alterations. By deciphering these diverse genetic and epigenetic markers, liquid biopsy enables researchers and clinicians to track tumor evolution and response to therapy, which will help in identifying potential therapeutic targets and stratifying patients for clinical trials.

Nevertheless, ongoing research actively addresses these limitations, aiming to improve the sensitivity, specificity, and comprehensiveness of liquid biopsy techniques. This continuous development paves the way for its even wider application in cancer diagnosis, treatment selection, and monitoring, ultimately leading to more personalized and effective cancer care [52].

Although various circulating biomarkers, such as cell-free RNAs, protein markers, and extracellular vesicles, hold promise, liquid biopsies are currently the brightest because of the use of ctDNA and CTCs. ctDNA, fragmented tumor DNA circulating in the blood, offers a noninvasive, real-time snapshot of tumor genomic alterations, aiding in diagnosis, prognosis, and monitoring treatment response. Its high abundance and dynamic nature enable tracking of tumor evolution and therapeutic efficacy. CTCs, which are rare tumor cells in the blood, represent the tumor itself, providing direct access to its genetic and phenotypic characteristics. The detection of these genes correlates with poor prognosis and treatment resistance, and their analysis can unveil potential therapeutic targets. However, both methods face limitations such as sensitivity and specificity to overcome these hurdles, pushing liquid biopsies toward becoming a cornerstone of personalized cancer management [53].

### Circulating tumor cells

Although the official discovery of circulating tumor cells (CTCs) in BC patient blood was not considered, the first report of their existence was by Thomas Ashworth in 1869 [54]. CTCs, which are generated from primary tumors and found in the bloodstream, hold considerable promise as biomarkers for cancer detection and monitoring [55]. By undergoing epithelial–mesenchymal transition (EMT), tumor cells transform into CTCs capable of entering the bloodstream. This critical step in metastasis has facilitated detailed tumor profiling in various studies, leading to improved prognostic capabilities [56]. However, what could be even more significant is that their use in diagnosing BC in the early stages could be even more significant. Various studies have reported the detection of CTCs in stage I to III BC patients [57, 58].

### ctDNA

Detecting MRD in cancer patients holds immense significance for guiding treatment decisions and predicting patient prognosis. In this context, ctDNA has emerged as a promising biomarker due to its ability to track the presence of tumor cells, even when it is undetectable by conventional methods [59].

## Characterizing ctDNA: unraveling its properties

The discovery of extracellular nucleic acid in human blood plasma in the late 1940s, which was initially identified in a patient with systemic lupus erythematosus, paved the way for the isolation of circulating tumor DNA (ctDNA) from blood samples from cancer patients due to advancements in technology [60]. This distinction is crucial, as healthy individuals primarily harbor cfDNA derived from hematopoiesis, while ctDNA reflects the presence of tumor-derived DNA fragments [61, 62].

Circulating tumor DNA has emerged as a game changer in cancer management. This "liquid biopsy" offers a minimally invasive window into the tumor’s inner workings, holding immense potential for improved disease monitoring, diagnosis, and personalized treatment selection. ctDNA levels act as a real-time reflection of tumor burden, allowing for early detection of recurrence and dynamic assessment of treatment response. This empowers clinicians to fine-tune treatment strategies and improve patient outcomes. Moreover, beyond confirming the presence of cancer, ctDNA analysis can provide valuable clues for early-stage detection, where conventional biopsies may be impractical. It holds promise for differentiating tumor types, pinpointing specific mutations, and guiding targeted therapy selection. This will help tailor treatment choices; that is, by revealing tumor-specific mutations and resistance mechanisms, ctDNA analysis empowers the development of personalized treatment plans. This approach maximizes treatment efficacy while minimizing side effects, suggesting the future of precision oncology. However, a deeper understanding of ctDNA efficacy and overcoming limitations in sensitivity and specificity remain active areas of research. Nevertheless, rapid advancements in ctDNA technology and its growing range of applications have solidified its position as a transformative force in cancer management [63–66].

Although identifying ctDNA biomarkers early remains challenging [64, 67], its analysis offers numerous advantages. ctDNA analysis can aid in the management of BC, particularly in patients where the disease has metastasized or in patients with early-stage recurrence after treatment. This minimally invasive "liquid biopsy" offers several key advantages: (1) Early recurrence detection, where in patients treated for early-stage disease, ctDNA allows for quicker and more sensitive detection of potential recurrence than traditional methods. This enables timely intervention and improved patient outcomes. (2) Personalized treatment selection occurs by identifying specific mutations in ctDNA genes to help clinicians tailor treatment strategies to the unique genetic profile of each patient's tumor.

This approach, known as precision oncology, allows for the selection of targeted therapies that are more likely to be effective while minimizing side effects. (3) Treatment monitoring, where tracking changes in ctDNA levels over time provides valuable insights into a patient's response to ongoing treatment. This allows for real-time adjustments to therapy plans, ensuring optimal effectiveness and avoiding unnecessary treatment burden; (4) Prognostic insights, as ctDNA analysis can reveal information about the tumor’s aggressiveness and potential for future progression, aiding in more informed prognostication and risk stratification; and (5) Understanding tumor heterogeneity and evolution, where ctDNA reflects the genetic diversity within a tumor, providing valuable details about its clonal evolution and potential therapeutic vulnerabilities. This information is crucial for developing effective treatment strategies that target the full spectrum of tumor subpopulations. Overall, ctDNA analysis empowers clinicians with a wealth of information that is essential for personalized management and improved outcomes in BC patients. While challenges in early detection remain an active area of research, the growing role of ctDNA across various stages of the disease underlines its immense potential for revolutionizing BC care [68–73].

### Amount of ctDNA

The amount of ctDNA detected in a patient's blood can vary depending on the location of the tumor. Studies have shown higher ctDNA levels in blood for certain cancers, including prostate, lung, colorectal, breast, and liver cancers [74]. Conversely, tumors such as gliomas and pancreatic, gastric, and oral cancers tend to shed lower amounts of ctDNA into the bloodstream [4].

### ctDNA vs. cfDNA

Despite holding immense potential for cancer diagnosis and monitoring, ctDNA typically comprises only a minuscule fraction (sometimes a mere 0.1%) of the total cell-free DNA (cfDNA) circulating in the bloodstream. This scarcity poses a significant challenge to its detection.

However, scientists have developed sophisticated methods that capitalize on specific hallmarks of ctDNA to distinguish it from the abundant cfDNA background. These hallmarks include somatic mutations, copy number variations (CNVs), aneuploidy, and DNA methylation. The choice of detection method depends on various factors, including the specific type of cancer, targeted genetic alterations, and desired sensitivity and specificity. Ongoing research strives to improve these methods further, enhancing their ability to detect even smaller amounts of ctDNA and enabling earlier cancer detection and personalized treatment strategies [49, 75].

**Fig. 4:**
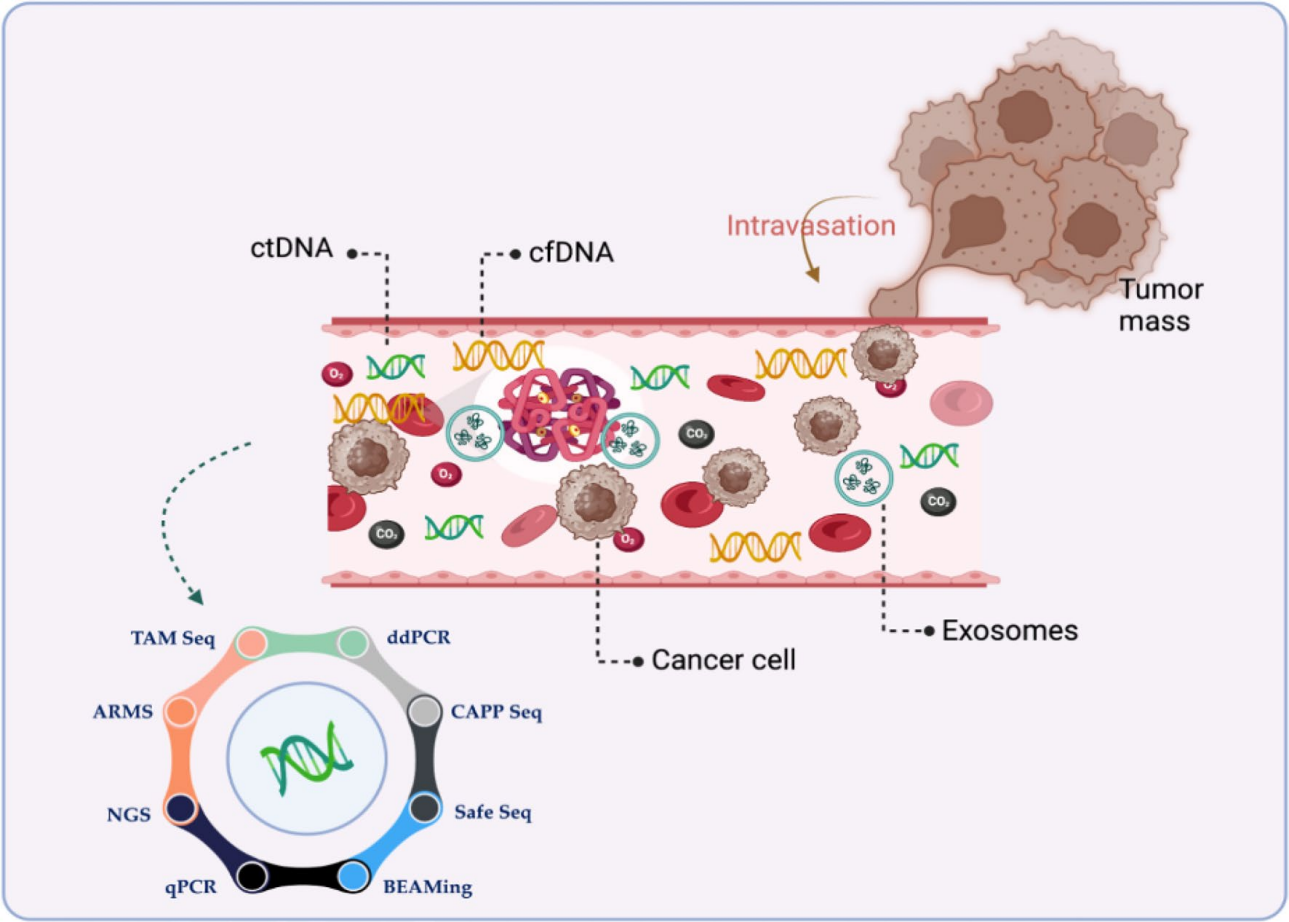
Tools that are used to detect circulating molecules to diagnose cancer. TAM-Seq: Targeted Amplification of Mutated Sequences, ARMS: Amplification Refractory Mutation System, NGS: Next- Generation Sequencing, qPCR: Quantitative Polymerase Chain Reaction, BEAMing: Bead-based Enrichment with Amplification and Magnetic Isolation, Safe SeqS: Safe Sequencing System, CAPP-Seq: Cancer Personalized Profiling by Deep Sequencing, and ddPCR: Droplet Digital PCR Created with BioRender

Each of them has a varying degree of sensitivity and specificity for detecting ctDNA in the presence of the more common cfDNA, depending on the method used and the intended clinical use. Somatic mutations are qualitative markers that distinguish ctDNA from cfDNA, while aneuploidy and copy number variation are quantitative markers. One copy of somatic mutations exists in each cell, and their distribution may be restricted, particularly in individuals with micrometastasis or early-stage disease. Different tools have been developed to detect cancer-related molecules circulating in the blood (Fig. 4). Table 1 highlights the pros and cons of each method.

**Table 1:** Circulating tumor DNA (ctDNA) detection methods

Method	Pros	Cons	References
Next-generation sequencing (NGS)	Unlike traditional methods that look at genes one at a time, NGS can scan millions of DNA snippets simultaneously. This allows it to: Spot tiny changes in genes (mutations): • These mutations can be single letters swapped, missing chunks, or even extra copies of genes, all of which can contribute to various diseases • Uncover rearrangements in chromosomes: Sometimes, pieces of chromosomes get flipped or swapped around, which can also impact health • See if genes are missing or duplicated: Having too many or too few copies of certain genes can also play a role in disease	• Traditional NGS strategies cannot detect variations with a frequency of less than 1.0% • High cost	[102–104]
Real-time quantitative PCR (qPCR)	It is commonly utilized for variant screening due to its high speed and low cost	It has a lower sensitivity (10%) than other detection methods	[105, 106]
Droplet digital PCR (ddPCR)	• ddPCR is more accurate and precise in rare target DNA (with 0.1% sensitivity for mutation) quantification than quantitative PCR • It can detect point mutation and short indels • When compared to tumor tissue, ddPCR exhibited accurate identification of PIK3CA mutations in patients with early-stage BC, with a sensitivity and specificity of 93.3% and 100%, respectively	• Currently, the ATRX mutation stands as a biomarker that cannot be effectively evaluated using ddPCR due to the widespread distribution of mutations throughout the entire gene • A significant drawback of the conventional ddPCR method is its limited ability to screen a finite number of mutations in each reaction, as it necessitates specific hydrolysis probes designed for each possible allelic variant. In contrast, an alternative approach known as drop-off ddPCR enhances throughput by utilizing only a single pair of probes to identify and measure potentially all genetic alterations within the targeted region	[107–114]
Amplification Refractory Mutation System (ARMS)	It is a quantitative PCR-based method that can detect < 1% mutant tumor tissue due to its high selectivity and sensitivity	• High cost of restriction enzymes • Time-consuming	[115, 116]
Beads, emulsions, amplification, and magnetic analysis (BEAMing)	• It is a simple, reliable, automatable, and sensitive test for detecting gene and transcript alterations • It could be used to measure other epigenetic changes, such as methylation, by treating DNA with bisulfite, which transforms methylated cytosine residues into thymidine • It can detect point mutation and short indels • High sensitivity of 0.02% detection rate	• The resulting aqueous compartment sizes were rather variable • Larger compartments are more likely to have multiple template molecules than small compartments • While this is not an issue for the analysis of rare variants, it is an issue if the variant to be analyzed is present in a significant proportion of the DNA molecules	[111, 117]
Tagged-Amplicon Sequencing (TAm-Seq)	• This approach has been extended to sequence, identify, and quantify tumor mutations across a group of genes, encompassing both hotspots in the tumor as well as complete coding sections of chosen genes • The detection limit for point mutations in EGFR in circulating DNA was 0.02%, and specificity was 99.99%, demonstrating the accuracy of this approach • TAm-seq has the potential to identify ~ 2% MAF (Mutant allele fraction) mutations with > 97% sensitivity	It could not identify low-frequency mutations in ctDNA and hotspots so it was developed to a new version enhanced TAm-Seq (eTAm-Seq™)	.[118, 119]
Safe-Sequencing System (Safe-SeqS)	• This method was developed to improve the sensitivity of NGS • It detects mutant alleles at concentrations as low as 0.1% and with 98.9% specificity • Safe-SeqS minimizes errors of sequencing by at least 70-fold and achieves up to ~ 98% sensitivity in detecting tumor mutations	• The drawback of this strategy is its reliance on detailed information about the tumor genome • However, targeted monitoring proves highly sensitive, quickly and inexpensively detecting variants with allele frequencies as low as 0.01% while maintaining a high level of accuracy	[120–123]
Personalized Profiling by Deep Sequencing (CAPP-Seq)	• This method enables highly specific and sensitive quantification of tumor-derived circulating tumor DNA (ctDNA), with a detection limit of approximately 0.01%. It is cost-effective and can be applied broadly to almost all types of tumors • CAPP-Seq method has the capability to detect various mutations in individuals with the same cancer type, allowing for the assessment of tumor heterogeneity. Previously, it has demonstrated the ability to identify tumor burdens before medical imaging. Moreover, it can identify a wide range of major mutation types, including insertions, deletions, single nucleotide variants, copy variants, and rearrangements • It is structured to control sequencing expenses by focusing on genomic regions that exhibit recurrent mutations • CAPP-Seq demonstrates its capability by identifying MAF at approximately 0.02% in stage II-IV NSCLC (non-small cell lung cancer) patients with an almost perfect sensitivity of nearly 100%	• Fusion mutations cannot be identified by CAPP-Seq • The detection limit of CAPP-Seq is affected by the input number and recovery rate of circulating DNA molecules, sample cross-contamination, potential allelic bias in the capture reagent, and PCR or sequencing errors	[124, 125]

Moreover, technical artifacts that cause background mutations and increase noise include PCR-based errors. Rearrangements and indels have the advantage of being detectable among millions of wild-type sequences, whereas point mutations are only found among thousands. This is because technical errors fail to generate specific rearrangements. Consequently, the signal-to-noise ratio is significantly greater than that with point mutations [4]. Translocations have limitations in that they are uncommon in solid tumors and are frequently unique to each patient, necessitating specialized testing. Methylation patterns in ctDNA have been applied to both the identification of cancers and the identification of the cancer's original tissue [76, 77].

One significant disadvantage is that molecular damage and loss during ctDNA template preparation before sequencing can restrict sensitivity, especially for applications requiring extreme sensitivity [78].

### ctDNA size

Beyond qualitative markers such as mutations, ctDNA and cfDNA exhibit a stark difference in size distribution. Research suggests that ctDNA fragments are enriched in a smaller size fraction than in a broader size range of cfDNA [79]. This phenomenon offers an exciting avenue for improving ctDNA detection by specifically analyzing mutations within this enriched fraction. However, maximizing this approach requires a comprehensive understanding of ctDNA properties, including its expected abundance in the sample, and careful consideration of the sensitivity and specificity limitations imposed by the chosen clinical application.

### ctDNA half-life

The half-life of ctDNA is the second most important characteristic for accurate detection. Numerous reports have shown that the half-life is not hours, days, or weeks but rather a few minutes. Pregnancy-related studies were the first to show that cfDNA typically has a short half-life. The fetal DNA was reduced by half on average in 16.3 min (range: 4–30 min). The fleeting nature of ctDNA, characterized by its short half-life, has emerged as a crucial factor for its accurate detection. Unlike expectations of hours, days, or weeks, numerous studies now reveal a surprisingly brief half-life for ctDNA, often measured in mere minutes. Pioneering research in pregnancy shed light on this phenomenon, demonstrating that fetal DNA in maternal blood, a form of cfDNA, exhibits rapid clearance with an average half-life of 16.3 min (ranging from 4 to 30 min). This finding sparked further investigations, confirming similarly short half-lives for ctDNA across various cancer types [80].

Understanding and accounting for this rapid turnover are essential for optimizing ctDNA detection strategies. The limited window of opportunity demands highly sensitive and rapid methods to capture ctDNA before it degrades and disappears from the bloodstream.

The short half-life of circulating tumor DNA (ctDNA) poses a significant challenge to its detection but also presents a unique opportunity for monitoring disease dynamics. Studies have revealed remarkably brief half-lives for ctDNA, with one report finding a median half-life of only 139 min for Epstein–Barr virus (EBV) DNA in plasma following surgical resection of nasopharyngeal cancer. Another study reported a similar 114-min half-life for ctDNA in a single patient with colorectal cancer after tumor removal [81].

This rapid turnover highlights the critical importance of highly sensitive and rapid detection methods for ctDNA. Given its potential association with both disease recurrence and treatment response, the disappearance and/or reappearance of ctDNA can serve as a powerful diagnostic indicator. Notably, a characteristic increase in ctDNA was detected immediately after surgical resection, followed by a rapid decrease. Subsequent increases in ctDNA levels may signal the presence of MRD, offering early warning of potential recurrence [81]. This underscores the utility of ctDNA as a dynamic biomarker for tracking tumor behavior and assessing treatment efficacy.

Since ctDNA can now be used to detect MRD and predict recurrence and response to therapy in a variety of tumor types before imaging modalities are available, ctDNA is an excellent biomarker for tracking dynamic changes in disease [82–84]. Table 1 provides a comprehensive overview of various ctDNA detection methods, highlighting their strengths and limitations.

## ctDNA and MRD in managing BC

The regular analysis of ctDNA is a powerful tool for monitoring BC progression and treatment response. This noninvasive approach, requiring only blood samples, opens avenues for early diagnosis, prognosis, and even predicting recurrence risk, as highlighted by Vlataki's work in 2023 [85].

Early detection remains the holy grail in BC, and ctDNA has immense potential due to its noninvasive nature. While challenging, several pilot studies, such as Zhang's in 2019, have shown promising results [86].

One such study by Rodriguez et al. (2019) explored the use of ctDNA analysis in patients with suspicious mammograms before resorting to tissue biopsies. Interestingly, 79.3% of the patients harbored TP53 mutations, and 34.5% had PIK3CA mutations, which mirrored their tumor profiles. Notably, one-third of the patients also exhibited plasma mutations, predominantly at very low levels. Additionally, patients with more severe clinical features displayed higher ctDNA levels, suggesting a potential link between disease severity and ctDNA abundance [87].

Furthermore, this study identified 13 total plasma mutations, 9 of which were associated with tissue mutations. Remarkably, 4 mutations (1 in PIK3CA and 3 in TP53) were solely detectable in plasma, indicating that tissue biopsies might miss crucial information. These “plasma-exclusive” mutations were even classified as driver mutations, highlighting the significant clinical implications of ctDNA analysis.

Although limited by the small sample size and focus on only two genes, this study underscores the potential of integrating ctDNA analysis into breast cancer screening. It also demonstrates the ability of ctDNA to capture tumor heterogeneity, providing valuable insights beyond traditional tissue biopsies [87].

Early detection and elimination of MRD are crucial goals for achieving higher cure rates for BC. As Cui (2023) noted, liquid biopsy can be used to predict BC metastasis and recurrence at early stages [88]. However, its relevance has expanded to solid tumors such as BC. These residual lesions, often undetectable by conventional imaging, can be identified using liquid biopsy. In addition to clinical indicators such as HER-2, ER, Ki-67, and PR, identifying distinct BC molecular subtypes can further refine the diagnosis and prognosis. As Abubakar (2019) mentioned, combining immunohistochemical analysis of these markers with the proliferation marker Ki-67 proves valuable in achieving this [89]. Stergiopoulou et al. conducted a study exploring the potential of liquid biopsy in BC management. They analyzed mutations in the ESR1 and PIK3CA genes and reported that one patient consistently lacked ESR1 mutations in plasma-derived ctDNA, suggesting their utility in monitoring disease status [90]. The significance of ctDNA analysis for BC is presented in Table 2.

**Table 2:** Significance of ctDNA analysis for BC clinical settings

BC detection at an early stage							
Stage	Targeted gene	Type of alteration	Detection method	Clinical utility	Pts number	Biological specimen	References
Early	TP53 PIK3CA	Point mutations	SafeSEQ	Diagnostic	29	Plasma	[87]
	TP53 NRAS CTNNB1 PIK3CA KPAS APC PTEN	Point mutations	NGS: Illumina	Diagnostic, screening	54	Plasma	[93]
	SFN P16 hMLH1 HOXD13 PCDHGB7 RASSF1a	DNA methylation	MethyLight	Diagnostic	749	Serum	[126]
	EGFR GREM1 PDGFRB PPM1E SOX17 WRN	DNA methylation	Microfluidic-PCR-based and NG	Diagnostic, screening	86	Plasma	[127]
	FHIT BRCA1	DNA methylation	BSP-HRMA	Diagnostic	66	Serum	[128]
	PIK3CA	Point mutations	ddPCR	Diagnostic	29	Plasma	[110]
	GSTP1 RASSF1A RARb2	DNA methylation	OS-MSP	Diagnostic	101	Serum	[129]
	APC GSTP1 RASSF1A RARbeta2	DNA methylation	qMSP	Diagnostic	93	Plasma	[130]

MRD detection and relapse prediction							
Stage	Targeted gene	Type of alteration	Detection method	Clinical utility	Pts number	Biological specimen	References
Early	PIK3CA TP53	Point mutations	WES	Prognostic	49	Plasma	[70]
	PIK3CA AKT PTEN	Point mutations	NGS: oncomine	Prognostic	38	Plasma	[131]
	PIK3R1 TP53 CDKN2A TP53	Point mutations	ddPCR	Prognostic	46	Plasma	[132]
	PIK3CA	Point mutations	ddPCR	Prognostic	55	Plasma	[101]
	GSTP1 RASSF1A RARb2	DNA methylation	OS-MSP	Prognostic	336	Serum	**.**[133]
	CST6	DNA methylation	MSP	Prognostic	27	Plasma	[134]
	BRCA1 MGMT GSTP1	DNA methylation	MSP	Prognostic	100	Serum	[135]
	RASSF1A	DNA methylation	MethyLight	Prognostic	148	Serum	[136]
Metastatic	TP53 ESR1 PIK3CA	Point mutations	NGS: Illumina	Prognostic	254	Plasma	[137]

Assessing the response to treatment or the development of resistance							
Stage	Targeted gene	Type of alteration	Detection method	Clinical utility	Pts number	Biological specimen	References
Early	PIK3CA ESR1	Point mutations	Multiplex ddPCR	Predictive	455	Plasma	[138]
	ESR1	Point mutations	ddPCR	Predictive	171	Plasma	[139]
	PR PROX MDGI PAX 5 RARb2	DNA methylation	MethDet-56	Predictive	20	Plasma	[140]
	ESR1	DNA methylation	MSP	Predictive	110	Plasma	[141]
	RASSF1A	DNA methylation	OS-MSP	Predictive	87	Plasma	[141]
	RASSF1A APC ADAM23 CXCL12	DNA methylation	Pyrosequencing	Predictive	34	Plasma	[142]
	RAS RASSF1A ESR1 CDH1 TIMP TIMP3 SYK	DNA methylation	multiplex MSP	Predictive	151	Plasma	[143]
Metastatic	KLK10 SOX17 WNT5A MSH2 GATA3	DNA methylation	MSP	Predictive	34	Plasma	[144]
	PIK3CA	Point mutations	BEAMing	Predictive	348	Plasma	[145]
	ESR1	Point mutations	ddPCR	Predictive	42	Plasma	[146]
	TP53 PIK3CA	Point mutations	WGS/ddPCR	Predictive	30	Plasma	[108]

## ctDNA: a game changer for breast cancer management—a path to personalized medicine

O'Leary et al. (2018) investigated tumor evolution in 195 BC patients treated with palbociclib and fulvestrant. They assessed ctDNA at treatment initiation and completion using a targeted amplicon panel for tumor purity evaluation and ddPCR for PIK3CA/ESR1 mutation detection [91].

Whole-exome sequencing (WES) of 14 patients revealed treatment-resistant subclonal mutations in RB1 and FGFR2. PyClone analysis and ddPCR confirmation further validated these findings. Leveraging WES data, they developed an error-corrected NGS panel for analyzing the remaining cohort, gaining insights into the genomic landscape and clonal evolution under this treatment regimen [92]. This combined approach of WES, targeted sequencing, and ddPCR provided a comprehensive understanding of tumor dynamics in this clinical setting.

### Integrating ctDNA with protein biomarkers

In addition to the use of ctDNA alone, integrating ctDNA with protein biomarkers holds promise for cancer detection. Notably, Cohen et al. [93] developed CancerSEEK, a multianalyte assay combining a 61-amplicon NGS panel for ctDNA quantification with an optimized protein biomarker panel for eight cancer types.

This combined approach achieved a median sensitivity of 70% and specificity exceeding 99% in detecting nonmetastatic cancers. Furthermore, machine learning-enabled tissue-of-origin prediction successfully identified primary tumors in more than 80% of patients across two anatomical sites.

Similarly, Cohen et al. [94] combined Safe-SeqS for KRAS mutations with a panel of protein biomarkers (CA-19-9, CEA, HGF, and OPN) for pancreatic cancer detection. This five-component assay yielded a sensitivity of 64%, significantly exceeding the 30% achieved by KRAS alone. These studies collectively demonstrate the synergistic power of combining ctDNA analysis with protein biomarkers, leading to enhanced sensitivity and potentially earlier cancer detection across various types.

The protein biomarker immunoassay platform was optimized for enhanced sensitivity while maintaining high specificity for detecting eight distinct cancer types (ovarian, liver, gastric, pancreatic, esophageal, colorectal, lung, and breast). In a study of 1005 patients with nonmetastatic cancer, CancerSEEK achieved a median sensitivity of 70% and a specificity exceeding 99%. Furthermore, machine learning-based tissue-of-origin prediction successfully identified the primary tumor in a median of 83% of patients across two anatomical sites and 63% at a single site [93].

These studies collectively showcase the synergistic power of combining noninvasive assays, leading to significantly enhancing cancer detection sensitivity.

### Personalized ctDNA analysis in chemotherapy response

In cases where common mutations are absent, personalized ctDNA analysis offers a promising alternative. This approach targets specific variants identified from the patient's primary tumor exome, potentially leading to earlier and more precise relapse detection [95].

Current chemotherapy regimens for BC, including neoadjuvant and adjuvant therapies, rely heavily on anthracyclines and taxanes. These drugs, while effective, have short- and long-term toxicity. Finding ways to adjust the dosage or duration of these treatments could hold the key to reducing these side effects while still improving overall survival rates. One promising approach involves using ctDNA to gauge treatment response more precisely than traditional radiological tests during neoadjuvant therapy. Unlike these tests, ctDNA offers greater sensitivity, potentially allowing for personalized treatment adjustments based on an individual's response [96].

Additionally, ctDNA analysis may identify patients who would not benefit from adjuvant chemotherapy, sparing them from unnecessary exposure to the drug's side effects. Furthermore, it could detect individuals with micro-metastases, indicating a greater risk of future distant metastases. This early detection allows for more targeted treatment plans, minimizing both overtreatment and the risk of future complications.

Therefore, ctDNA analysis holds immense potential to improve patient selection for chemotherapy regimens in breast cancer patients. Offering a more sensitive and personalized approach to treatment response assessment could pave the way for better outcomes with fewer side effects [95].

Identifying patients with early BC who truly benefit from adjuvant therapy remains a critical challenge. While traditional treatments such as chemotherapy and endocrine therapy are effective for many patients, a smaller group experiences unnecessary side effects without any long-term survival benefit. This highlights the need for biomarkers to detect MRD (micro-metastases) and tailor treatment accordingly. ctDNA analysis has emerged as a promising tool in this context. However, ctDNA analysis in early BC patients faces difficulties due to the typically low levels of ctDNA shed by micro-metastases. Despite these challenges, recent studies employing highly sensitive digital droplet PCR (ddPCR) have shown promising results. These studies demonstrate that postsurgical ctDNA levels can quantitatively predict both poor prognosis and the risk of relapse. This suggests that ctDNA analysis could help identify patients who truly need adjuvant therapy, sparing others from unnecessary risks and potential harm [97, 98].

Detecting and monitoring minimal residual disease (MRD) is crucial for assessing treatment response and informing subsequent therapeutic decisions. The presence of PIK3CA or TP53 mutations before neoadjuvant therapy is linked to a lower rate of pathological complete response (pCR), suggesting that individuals with these mutations may benefit from a more aggressive or targeted treatment approach [99]. Another study revealed that the presence of mutant PIK3CA ctDNA before surgery was associated with poor relapse-free survival and overall survival, independent of the BC subtype. High ctDNA levels before neoadjuvant treatment are correlated with larger tumor size, increased aggressivity, and specific subtypes.

Detecting and monitoring MRD is essential for accurately assessing a patient's response to treatment and guiding subsequent therapeutic decisions. This plays a crucial role in personalizing cancer treatment and improving patient outcomes. Studies have shown that the presence of specific genetic mutations, such as PIK3CA or TP53, before treatment can predict a lower rate of complete tumor elimination (pathological complete response, pCR). This suggests that individuals with these mutations may benefit from more aggressive or targeted therapies than from standard approaches [99].

Furthermore, research indicates that the presence of mutant PIK3CA circulating tumor DNA (ctDNA) before surgery is associated with poorer outcomes, including shorter relapse-free survival and overall survival, regardless of the specific breast cancer subtype. Additionally, high levels of ctDNA before treatment are linked to larger tumors, increased tumor aggressiveness, and specific cancer subtypes.

These findings highlight the importance of considering MRD and specific genetic mutations when tailoring treatment plans for cancer patients. By incorporating this information, doctors can potentially improve treatment effectiveness and optimize patient outcomes.

A fascinating finding in cancer research is the link between circulating tumor DNA (ctDNA) and neoadjuvant treatment outcomes. Specifically, the presence of ctDNA after treatment is associated with lower rates of pathological complete response (pCR), a measure of complete tumor eradication. Conversely, the clearance of ctDNA after treatment predicts longer survival, even for patients who do not achieve pCR [72].

In 2019, a significant milestone was achieved in the diagnosis and treatment of advanced breast cancer (ABC) with the approval of plasma-extracted ctDNA as a method to select patients for targeted therapy [100]. This marked the first time a liquid biopsy companion diagnostic was used for drug approval, paving the way for more personalized and effective treatment approaches.

**Fig. 5:**
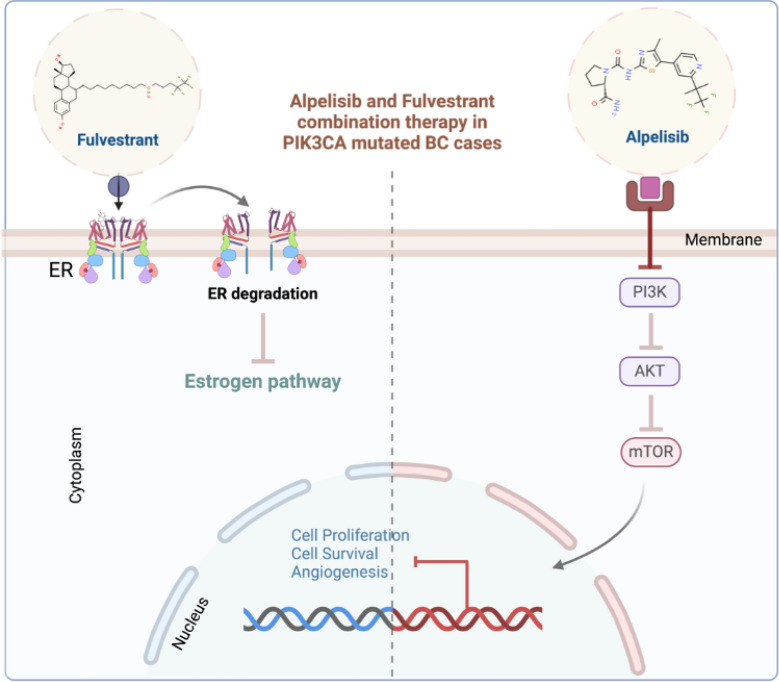
Precision medicine for BC. Tailoring breast cancer treatment with alpelisib and fulvestrant. For patients with breast cancer harboring specific PI3K mutations, precision medicine is promising. Alpelisib, a targeted therapy, combines with Fulvestrant, a hormonal agent, to deliver a powerful one-two punch in the adjuvant setting. This personalized approach not only attacks the unique vulnerabilities of cancer patients but also extends progression-free survival, potentially delaying the need for further treatment and offering a brighter outlook for these individuals. This targeted strategy represents a significant step forward in the fight against breast cancer, ushering in an era where treatment aligns with a patient's specific molecular profile, maximizing efficacy while minimizing unnecessary side effects. Created with BioRender

Alpelisib, an oral alpha-selective PIK3CA inhibitor, became the first drug to benefit from this innovative approach. Combined with fulvestrant, it specifically targets HR-positive, HER2-negative ABC patients harboring PIK3CA mutations. This targeted therapy was made possible thanks to a rigorous development process that began with a phase I study. This initial study evaluated the safety and efficacy of alpelisib in combination with fulvestrant for treating ER-positive advanced BC patients, regardless of their PIK3CA mutation status. The subsequent identification of PIK3CA mutations as key drivers of response in this patient subgroup led to the development of ctDNA-based companion diagnostic methods and targeted approval for PIK3CA-mutated patients (Fig. 5).

Researchers found that a targeted therapy combination significantly improved outcomes for patients with a specific genetic mutation in their cancer. Patients with PIK3CA mutations who received alpelisib and fulvestrant had a 29% response rate compared to patients without PIK3CA mutations who did not respond. Additionally, the progression-free survival (time before the cancer worsened) was nearly double that in the PIK3CA-mutated group, at 9.1 months compared to 4.7 months. This personalized approach using genetic testing to guide treatment decisions holds promise for improving outcomes in BC patients [46].

By analyzing 455 breast cancer patients, researchers found that 15.2% had mutations in the PIK3CA and/or TP53 genes. They tracked ctDNA throughout treatment and found that it was most prevalent before treatment (41%). Patients who received ctDNA pretreatment were older, had estrogen receptor-negative tumors, and were 85% less likely to achieve complete tumor eradication. Although its impact on long-term survival remains unclear, this study suggested that ctDNA could predict treatment response. Notably, patients with HER2-positive tumors lacking early ctDNA had the highest pCR rates, while those with persistent ctDNA fared the worst. This highlights the potential of ctDNA analysis for personalized treatment approaches and identifying high-risk patients [99]

A study of 55 early-stage BC patients revealed that ctDNA could predict relapse after curative therapy. Compared with traditional methods, monitoring mutations in ctDNA samples over time increased the prediction accuracy by 7.9 months.

Remarkably, this liquid biopsy approach identified MRD-related genetic events that predicted future metastatic recurrence more accurately than analyzing the original tumor. This suggests that tracking mutations in ctDNA could identify high-risk patients and guide personalized treatment, potentially overcoming the obstacle of tumor genetic diversity [101]. While numerous resistance-linked mutations have been identified, further research is needed to translate this knowledge into targeted therapies, paving the way for even more precise and effective interventions [98]

## Clinical trials

Our review analyzed 40 ongoing clinical trials on ClinicalTrials.gov that explored the potential of liquid biopsy methods, specifically circulating tumor DNA (ctDNA), for breast cancer (BC) treatment (Table 3). Liquid biopsy goes beyond traditional methods, investigating its role in early detection, personalized treatment decisions (both interventional and prognosis), and ongoing patient monitoring. Research has even explored combining liquid biopsy with imaging techniques such as PET-CT. Additionally, studies have compared how liquid biopsy function in early versus metastatic stages across different types of luminal BC (Table 3). These promising findings suggest that ctDNA analysis could become a key element in personalized BC management, impacting everything from early detection to long-term care.

**Table 3:** Clinical trials of breast ctDNA-based ctDNA-based MRD detection

Trial registration	Study Status	Design	Study type	Intervention/observational model	Country	Pt number	ctDNA detection method	Study outcomes	Refs.
NCT04920708	Recruiting	Randomized	Interventional	Parallel Assignment	UK	324	–	Assessing the progression-free survival (PFS) in advanced ER + /HER2- BC patients carrying identifiable mutations and exhibiting high levels of ctDNA after two weeks of CDK4/6 inhibitor/fulvestrant treatment, aiming to contrast the PFS among those randomly assigned to receive palbociclib/fulvestrant/ipatasertib with those receiving the standard palbociclib-fulvestrant treatment	[147]
NCT05858242	Recruiting	–	Observational	Cohort	China	1000–1050	plasma ctDNA polygene methylation test	• Identifying the ctDNA methylation targets that are specific to BC • Examine the use of postoperative ctDNA methylation in assessing the prognostic value of BC surgery and subsequent surveillance	[148]
NCT06087120	Recruiting	–	Observational	Cohort	Vietnam	–	PCR based techniques	determine the prevalence and evolution of ctDNA in cancer patients' blood samples before, during, and following neoadjuvant therapy Examining how pCR response in neo-adjuvant therapy relates to ctDNA expression on MRI imaging	[149]
NCT03079011	Active, not recruiting	Randomized	Interventional	Sequential Assignment	France	1017	–	• Incidence of adverse events that arise during treatment • Both progression-free and chemotherapy-free survival are possible	[150]
NCT05333874	Active, not recruiting	Nonrandomized	Interventional pilot study	Single Group Assignment	United States	34	PCR based techniques	Levels of Detectable ctDNA	[151]
NCT04308720	Recruiting	–	Observational	Cohort	United States	400	–	• Variations in the mutational burden and ctDNA detection rate in BC patients who show indications of regional nodal irradiation • The alteration in the percentage of patients exhibiting detectable ctDNA after treatment and three months afterward in comparison to the initial measurement • The relationship between detectable ctDNA at each specific time point and the survival period without recurrence or invasion	[152]
NCT05945290	Recruiting	–	Observational	Cohort	United States	120	TARgeted DIgital Sequencing (TARDIS) and Quality Assessment (QA) assay	Variation in the levels of tumor-specific circulating tumor DNA (ctDNA)	[153]
NCT05770531	Recruiting	Randomized	Interventional	Parallel Assignment	United States	120	–	PFS and OS	[154]
NCT04354064	Recruiting	–	Observational	Cohort	United States	3362	–	Freedom from progression and Overall survival (OS)	[155]
NCT04872608	Withdrawn	N/A	Interventional	Single Group Assignment	United States	–	–	The recommended dose for phase 2 (RP2D) of onapristone ER	[156]
NCT04768426	Recruiting	N/A	Interventional	Single Group Assignment	United States	25	next-generation sequencing	Initial ctDNA detection levels, ctDNA levels, tumor genomic feature correlation, overall survival (OS), and relapse-free survival	[157]
NCT03318263	Completed	N/A	Interventional	Single Group Assignment	France	146	NGS	• Incidence of mutations in ESR1 • Incidence of PIK3CA and AKT1 mutations • Prevalence of mutations in AKT1, PIK3CA, and ESR1 in patients with and without endocrine resistance at enrollment • The frequency of AKT1, PIK3CA, and ESR1 mutations in patients depending on whether they underwent monotherapy or a combination of treatments • Prevalence of mutations from the initiation of treatment until the end of the follow-up period or progression in other noteworthy genes within the panel • Mutations in AKT1, PIK3CA, and ESR1 can be used to predict survival without progression	[158]
NCT05099978	Recruiting	–	Observational	Cohort	Japan and Malaysia	506	NGS	• Percentage of patients among all examination cases that had one or more genetic abnormalities • To report the concordance rate, the genomic anomalies of the tumor tissue and ctDNA will be merged	[159]
NCT03145961	Active, not recruiting	Randomized	Interventional	Parallel Assignment	United Kingdom	208	–	12 months after the positive ctDNA finding Positive ctDNA detection after a full year The lack of observable ctDNA or any sign of disease recurrence after six months (24 weeks) from the commencement of pembrolizumab treatment	[160]
NCT05428709	Terminated	N/A	Interventional	Single Group Assignment	United States	3	–	Changes in TGF-β, VEGF-A, S1P, TSP-1, and ctDNA levels	[161]
NCT04567420	RECRUITING	Randomized	Interventional	Parallel Assignment	United States	100	–	Clinically obvious metastatic or local BC is associated with positive ctDNA findings Examine if a positive ctDNA result indicates a clinical recurrence	[162]
NCT03947736	–	–	Observational	Cohort	China	200	ddPCR, and NGS	• Investigation of the correlation between the abundance of a particular mutation or plasma HER2 amplification and imaging evaluation in Individuals diagnosed with metastatic or recurrent BC • Examine the extent to which plasma HER2 ctDNA can predict therapy efficacy for recurrent or metastatic BC	[163]
NCT05814224	Recruiting	N/A	Interventional	Single Group Assignment	Italy	164	–	• Luminal BC therapy response is tracked by liquid biopsy • miRNA/ctDNA-based surveillance • Time to Progression (TTP), and PFS	[164]
NCT05625087	Recruiting	Randomized	Interventional	Parallel Assignment	France	162	–	• Survival without progression and the overall survival rate of the entire study group • To validate that individuals who were randomly assigned and had residual PIK3CA on their ctDNA had worse outcomes than patients who were not randomly assigned	[165]
NCT03017573	Recruiting	N/A	Interventional	Single Group Assignment	France	700	–	• Relationship between baseline clinic biological characteristics and the molecular/immunological profile of the tumor • Correlation between the immune system, de novo ctDNA mutations, and ctDNA levels	[166]
NCT03285412	Recruiting	Randomized	Interventional	Parallel Assignment	United States	120	–	ctDNA clearance	[167]
NCT05050890	–	–	Observational	Cohort	Brazil	38	–	• ctDNA concentration • Change from the beginning of the neoadjuvant treatment until the end of the ctDNA	[168]
NCT02546232	Active, not recruiting	Randomized	Interventional	Parallel Assignment	Norway	196	–	Cohorts I and II exhibit varying degrees of pathologic complete response (pCR), partial response (PR), complete response (CR), progressive disease (PD), and stable disease (SD) in treatment response Circulating tumor-DNA in plasma	[169]
NCT04256941	Completed	Randomized	Interventional	Parallel Assignment	United States	4	scatter plots	• ESR1 mutant allele fraction (MAF) and dynamics of circulating tumor deoxyribonucleic acid (ctDNA) • Prevalence of Estrogen Receptor 1 (ESR1) mutation emergence • Alterations in the tumor marker CA 15–3 cancer antigens • OS and PFS	[170]
NCT01957332	ACTIVE, NOT RECRUITING	N/A	Interventional	Single Group Assignment	Netherlands	217	–	Linkage between imaging, molecular analysis, and subsequent data with the analysis of DNA, miRNA, peptides, and RNA	[171]

## Conclusions and future perspectives

The domain of breast cancer (BC) management is currently undergoing a pivotal transformation, greatly propelled by advancements in plasma-based liquid biopsy research. This burgeoning field illuminates the intricate utility of ctDNA and other molecular markers, not only as tools for prognostication but also as pivotal elements in guiding therapeutic decisions. The analysis of ctDNA has transitioned from a conceptual framework to a practical clinical tool, offering insights into tumor dynamics, resistance mechanisms, and MRD. As evidenced by numerous active clinical trials, the potential of ctDNA in enhancing the precision of care in both the early and advanced stages of BC is becoming increasingly tangible.

However, this journey is not devoid of challenges. The path to integrating ctDNA and MRD analysis into standardized BC management is fraught with technical and methodological obstacles. Issues such as the optimization of ctDNA extraction and the need for harmonization in detection techniques are paramount. Furthermore, the quest for robust, large-scale, prospective studies is critical to establish the unequivocal clinical impact of ctDNA, ensuring that its application is grounded in solid evidence, enhancing patient outcomes across all spectra of the disease.

Looking ahead, BC management's horizon is set to expand beyond the realms of ctDNA. A comprehensive, integrative approach that encompasses a spectrum of biomolecules, including cell-free RNA, extracellular vesicles, circulating tumor cells, and microRNAs, is proposed to revolutionize our understanding of this disease. This multifaceted strategy promises to unravel the complexities of the tumor microenvironment, offering a panoramic view of cancer biology and its evolutionary trajectory.

A notable advancement in this direction is the CancerSEEK technique, which synergizes genetic and protein biomarkers to accurately identify the tissue of origin in early-stage cancers. This innovation exemplifies the potential of integrating liquid biopsy into the fabric of routine clinical management, potentially transforming early detection and treatment paradigms.

Furthermore, the integration of artificial intelligence and machine learning algorithms for analyzing liquid biopsy data holds promise for identifying novel biomarkers, predicting treatment responses, and personalizing patient care. As we move forward, collaborative efforts between clinicians, researchers, and technologists will be crucial in translating these innovations from bench to bedside, ensuring that the future of BC management is not only about treating the disease but also preempting it, personalizing interventions and ultimately improving the quality of life for patients worldwide.

## Data Availability

No datasets were generated or analysed during the current study.

## Abbreviations

ABC: Advanced breast cancer
ADAM23: ADAM metallopeptidase domain 23
AI: Aromatase inhibitor
AKT: Serine/threonine kinase 1
APC: Adenomatous polyposis coli
ARMS: Amplification refractory mutation system
ATRX: Alpha-thalassemia/mental retardation, X-linked
BEAMing: Beads, emulsions, amplification, and magnetic analysis
BRCA: Breast cancer gene
BSP-HRMA: Bisulfite sequencing method and high-resolution melting curve analysis
CA: Carbohydrate antigen
CAPP-Seq: Personalized profiling by deep sequencing
CDH1: Cadherin 1
CDKN2A: Cyclin-dependent kinase inhibitor 2A
CEA: Carcinoembryonic antigen
cfRNA: Circulating free DNA
CK: Cytokeratin
CR: Complete response
CRC: Colorectal cancer
CST6: Cystatin M
CTCs: Circulating tumor cells
ctDNA: Circulating tumor DNA
CTNNB1: Catenin beta-1
CXCL12: C-X-C Motif Chemokine Ligand 12
ddPCR: Droplet digital polymerase chain reaction
dPCR: Digital polymerase chain reaction
EBV: Epstein-barr virus
EFS: Event-free survival
EGFR: Epidermal growth factor receptor
ER: Estrogen receptor
ESR1: Estrogen receptor 1
EVs: Extracellular vesicles
FGFR: Fibroblast growth factor receptor
FHIT: Fragile histidine triad diadenosine triphosphatase
FISH: Fluorescence in situ hybridization
GATA3: GATA binding protein 3
GPCRs: G-protein-coupled receptors
GREM1: Gremlin 1
GSTP1: Glutathione S-transferase Pi 1
HER2: Epidermal growth factor receptor 2
HGF: Hepatocyte growth factor
hMLH1: Human mutL homolog 1
HOXD13: Homeobox D13
IHC: Immunohistochemical
KLK10: Kallikrein Related Peptidase 10
KPAS: Keratosis pilaris atropican
LB: Liquid biopsies
MAF: Mutant allele fraction
MDGI: Mammary-derived growth inhibitor
MES: Claudin-low, mesenchymal,
MethDet: Methylation detection,
MGMT: O6-methylguanine-DNA methyltransferase
MRD: Minimal residual disease
MSH2: MutS homolog 2
MSP: Methylation-specific polymerase chain reaction
MUC4: Mucin antigen 4
ncRNA: Noncoding RNA
NGS: Next generation sequencing
NRAS: Neuroblastoma RAS viral oncogene homolog
NSCLC: Non-small cell lung cancer
OPN: Osteopontin
OS: Overall survival
OS-MSP: One-step methylation-specific polymerase chain reaction
P16: Cyclin-dependent kinase inhibitor 2A
PAX 5: Paired box gene 5
PCDHGB7: Protocadherin gamma subfamily B7
pCR: Pathological complete response
PD: Progressive disease
PDGFRB: Platelet-derived growth factor receptor beta
PDK1: Phosphoinositide-dependent protein kinase 1
PET-CT: Positron emission tomography-computed tomography Scan
PFS: Progression-free survival
PH: Pleckstrin homology
PI3K: Phosphatidylinositol 3-kinase
PI3Kα: Phosphoinositide 3-kinase alpha
PIK3CA: Phosphatidylinositol-4,5-bisphosphate 3-kinase catalytic subunit alpha
PIK3R1: Phosphoinositide-3-kinase regulatory subunit 1
PIP2: Phosphatidylinositol 4,5-bisphosphate
PIP3: Phosphatidylinositol (3,4,5)-trisphosphate
PPM1E: Protein Phosphatase, Mg2 + /Mn2 + Dependent 1E
PR: Progesterone receptor
PTEN: Phosphatase and tensin homolog
PTMs: Histone posttranslational modification
QA: Quality assessment assay
qMSP: Quantitative methylation-specific PCR
qPCR: Real-time quantitative PCR
RARbeta2: Retinoic acid receptor beta
RAS: Rat sarcoma
RASSF1a: RAS association domain family protein 1a
RP2D: The recommended dose for phase 2
RTKs: Receptor tyrosine kinases
S1P: Sphingosine 1-phosphate
Safe-Seq: Safe-sequencing
Safe-SeqS: Safe-Sequencing System
SD: Stable disease
SFN: Stratifin
SNPs: Single nucleotide polymorphisms
SOX17: SRY-box transcription factor 17
SYK: Spleen tyrosine kinase
TAm-Seq: Tagged-amplicon sequencing
TARDIS: Targeted digital sequencing
TGF-β: Transforming growth factor-beta
TIMP: TIMP metallopeptidase inhibitor
TNBCs: Triple-negative breast cancers
TP53: Tumor protein p53
TSP-1: Thrombospondin-1
TTF: Longer time to treatment failure
TTP: Time to progression
VAF: Variant allele frequency
VEGF-A: Vascular endothelial growth factor A
WES: Whole exome sequencing
WGS: Whole genome sequencing
WNT5A: Wnt family member 5A
WRN: Werner syndrome helicase
